# Increased risk of asthma in patients with rheumatoid arthritis: A longitudinal follow-up study using a national sample cohort

**DOI:** 10.1038/s41598-019-43481-3

**Published:** 2019-05-06

**Authors:** So Young Kim, Chanyang Min, Dong Jun Oh, Hyo Geun Choi

**Affiliations:** 1 Department of Otorhinolaryngology-Head & Neck Surgery, CHA Bundang Medical Center, CHA University, Seongnam, Korea; 2 Hallym Data Science Laboratory, Hallym University College of Medicine, Anyang, Korea; 3 Graduate School of Public Health, Seoul National University, Seoul, Korea; 4 Department of Otorhinolaryngology-Head & Neck Surgery, Hallym University College of Medicine, Anyang, Korea; 5 Department of Internal medicine, Asan Medical Center, University of Ulsan College of Medicine, Seoul, Korea

**Keywords:** Asthma, Rheumatoid arthritis

## Abstract

The aim of this study was to evaluate the risk of asthma in rheumatoid arthritis patients using matched control group for socioeconomic factors and past medical history. Adults >20 years old were collected from the Korean Health Insurance Review and Assessment Service - National Sample Cohort (HIRA-NSC) from 2002 through 2013. A total of 6,695 individuals with rheumatoid arthritis were matched for age, sex, income, region of residence, hypertension, diabetes, and dyslipidemia with 26,780 individuals included in a control group. In both the rheumatoid arthritis and control groups, subjects’ history of asthma was evaluated. Asthma (J45 and J46) and rheumatoid arthritis (M05 and M06) were included based on the International Classification of Disease-10 (ICD-10) codes and medication history. The crude and adjusted (depression and Charlson Comorbidity Index) hazard ratios (HRs) and 95% confidence intervals (CI) of asthma for rheumatoid arthritis patients were analyzed using a stratified Cox proportional hazard model. Subgroup analyses were conducted according to age and sex, number of treatment histories, and medication histories. Approximately 16.4% (1,095/6,695) of rheumatoid arthritis group and 13.0% (3,469/26,780) of the control group had asthma (P < 0.001). The rheumatoid arthritis group demonstrated a higher adjusted HR for asthma than the control group (adjusted HR = 1.23, 95% CI = 1.15–1.32, P < 0.001). This result was consistent in all subgroups. Rheumatoid arthritis was related to an increase risk of asthma.

## Introduction

Rheumatoid arthritis is an autoimmune disease that manifests as a synovial inflammation, hyperplasia and joint, cartilage and bone destruction^1^. The prevalence of rheumatoid arthritis is varies according to ethnicity and residential region from approximately 0.48–1% in adult population^2^. In Korea, approximately 0.27% of the general population has rheumatoid arthritis^3^. Rheumatoid arthritis has been reported to increase the risk of several morbidities including acute myocardial infarction, chronic obstructive pulmonary disease and asthma^4–6^. Thus, considerable attention has been paid to minimize the burden of rheumatoid arthritis by elucidating associated factors. The etiology of rheumatoid arthritis is yet to be completely elucidated, and multiple factors including genetic and environmental triggers and immunologic factors are known to be involved in the pathophysiology of rheumatoid arthritis^1^. Because of these multiple causative factors, several systemic diseases, such as cardiovascular disease and diabetes, have been described to be related to rheumatoid arthritis^7,8^.

Asthma is a chronic inflammatory disease of the airway with a heterogeneous pathophysiology^9,10^. The prevalence of asthma is approximately 4.3–8.6% in the adult population globally^11,12^. In Korea, approximately 5.7% of adults have asthma^13^. In addition to causing well-known pathologies in the airway, several studies have proposed systemic inflammatory dysfunction in asthma patients^14,15^. In addition to generating T helper cell type 2 (Th2) immune responses, Th1-predominant inflammatory factors such as tumor necrosis factor (TNF)-α are elevated in asthma patients^16^. In line with this, other inflammatory diseases, such as rheumatoid arthritis, have been suggested to be associated with asthma^5,17–19^. A cross-sectional study reported a positive association between rheumatoid arthritis and asthma (adjusted odds ratio [OR] = 3.12, 95% confidence interval [CI] = 2.77–3.51)^19^. However, a causal relationship between asthma and rheumatoid arthritis could not be determined due to the cross-sectional study design. A few prior longitudinal follow-up studies reported an increased risk of rheumatoid arthritis in asthma patients^20,21^. On the other hand, only one recent population cohort study described a high risk of asthma in rheumatoid arthritis patients^5^.

The running hypothesis of the present study was that rheumatoid arthritis could increase the risk of asthma in an adult population independent of demographic factors and patients’ past medical history. To prove this hypothesis, we used a large, population cohort database of medical claim codes. To minimize possible bias from medical accessibility, participants’ level of income and region of residence were additionally matched for in the control group. Moreover, participants’ past medical history of hypertension, diabetes, and dyslipidemia were matched between the study and control groups.

## Results

The time duration from index date to asthma was 45.6 months (Standard deviation, SD = 34.5) in asthma group and 46.2 months (SD = 35.1) in control group. The rate of asthma was higher in the rheumatoid arthritis group (16.4% [1,095/6,695]) than in the control group (13.0% [3,469/26,780], P < 0.001, Table 1). The general characteristics (age, sex, income, region of residence, and hypertension, diabetes, and dyslipidemia) of the participants were the same due to the matching procedure (P = 1.000). The rates of depression, and Charlson Comorbidity Index (CCI) score ≥2 were higher in the rheumatoid arthritis group than in the control group (each P < 0.05).

**Table 1:** General Characteristics of Participants.

Characteristics	Total participants		
	Rheumatoid arthritis (n, %)	Control (n, %)	P-value
Age (years old)			1.000
20–24	176 (2.6)	704 (2.6)	
25–29	281 (4.2)	1,124 (4.2)	
30–34	382 (5.7)	1,528 (5.7)	
35–39	509 (7.6)	2,036 (7.6)	
40–44	667 (10.0)	2,668 (10.0)	
45–49	914 (13.7)	3,656 (13.7)	
50–54	1,059 (15.8)	4,236 (15.8)	
55–59	828 (12.4)	3,312 (12.4)	
60–64	767 (11.5)	3,068 (11.5)	
65–69	548 (8.2)	2,192 (8.2)	
70–74	339 (5.1)	1,356 (5.1)	
75–79	168 (2.5)	672 (2.5)	
80–84	49 (0.7)	196 (0.7)	
85+	8 (0.1)	32 (0.1)	
Sex			1.000
Male	1,569 (23.4)	6,276 (23.4)	
Female	5,126 (76.6)	20,504 (76.6)	
Income			1.000
1 (lowest)	1,077 (16.1)	4,308 (16.1)	
2	979 (14.6)	3,916 (14.6)	
3	1,195 (17.8)	4,780 (17.8)	
4	1,516 (22.6)	6,064 (22.6)	
5 (highest)	1,928 (28.8)	7,712 (28.8)	
Region of residence			1.000
Urban	2,952 (44.1)	11,808 (44.1)	
Rural	3,743 (55.9)	14,972 (55.9)	
Hypertension	2,829 (42.3)	11,316 (42.3)	1.000
Diabetes	1,387 (20.7)	5,548 (20.7)	1.000
Dyslipidemia	2,272 (33.9)	9,088 (33.9)	1.000
Depression	859 (12.8)	2,740 (10.2)	<0.001*
CCI (score)			<0.001*
0	2,061 (30.8)	11,988 (44.8)	
1	1,036 (15.5)	3,602 (13.5)	
≥2	3,598 (53.7)	11,190 (41.8)	
Asthma ≥2 times	1,095 (16.4)	3,469 (13.0)	<0.001*
Asthma ≥3 times	786 (11.7)	2,435 (9.1)	<0.001*
Asthma ≥4 times	626 (9.4)	1,862 (7.0)	<0.001*
Asthma ≥5 times	500 (7.5)	1,477 (5.5)	<0.001*

^*^Chi-square test or Fisher’s exact test. Significance at P < 0.05.

CCI: Charlson Comorbidity Index.

**Figure 1:**
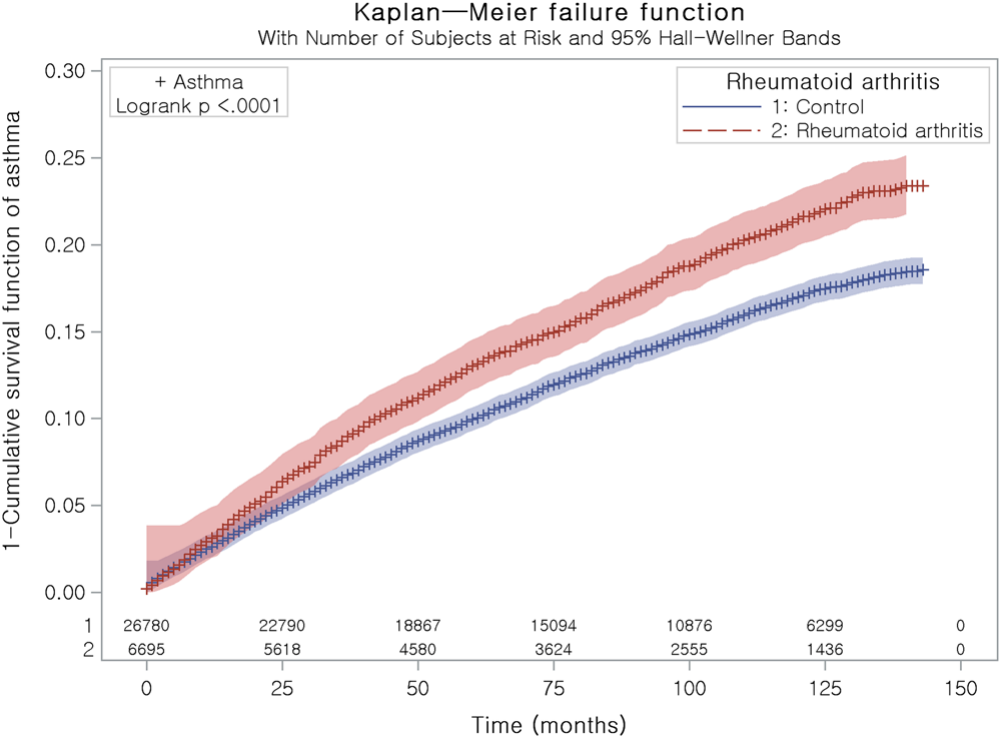
Kaplan Meier curve of rheumatoid arthritis for asthma. It was explained as 1 – survival function curve.

The adjusted HR of asthma was 1.23 in the rheumatoid arthritis group compared to the control group (95% CI = 1.15–1.32, P < 0.001, Table 2 and Fig. 1).

**Table 2:** Crude and adjusted hazard ratios (95% confidence interval) of rheumatoid arthritis for asthma.

Characteristics	Asthma			
	Crude^†^	P-value	Adjusted^†,‡^	P-value
Rheumatoid arthritis	1.31 (1.22–1.40)	<0.001*	1.23 (1.15–1.32)	<0.001*
Control	1.00		1.00	

^*^Cox-proportional hazard regression model, Significance at P < 0.05.

^†^Stratified model for age, income, and region of residence, hypertension, diabetes mellitus, and dyslipidemia histories.

^‡^Adjusted model for depression histories, and Charlson Comorbidity Index.

In subgroup analyses performed according to age and sex, all the crude and adjusted HRs of asthma were higher in the rheumatoid arthritis group than in the control group (each of P < 0.05, Table 3). The adjusted HRs were 1.61 (95% CI = 1.06–2.45) in men <40 years old; 1.40 (95% CI = 1.15–1.71) in women <40 years old; 2.20 (95% CI = 1.71–2.82) in men 40–59 years old; 1.16 (95% CI = 1.05–1.29) in women 40–59 years old; 1.47 (95% CI = 1.13–1.93) in men ≥60 years old; and 1.19 (95% CI = 1.05–1.35) in women ≥60 years old. According to the number of treatment histories of asthma, the rate of ≥3, ≥4, and ≥5 times of treatment histories of asthma were elevated in rheumatoid arthritis patients (all P < 0.001, Table 4). According to the types of medications, rheumatoid arthritis patients who treated with methotrexate, lefunomide, and other medications demonstrated 1.27 (95% CI = 1.14–1.43), 1.23 (95% CI = 1.02–1.48), and 1.20 (95% CI = 1.09–1.32) higher adjusted HRs for asthma (Table 5).

**Table 3:** Subgroup analysis of crude and adjusted hazard ratios (95% confidence interval) of rheumatoid arthritis for asthma according to age and sex.

Characteristics	Asthma			
	Crude^†^	P-value	Adjusted^†,‡^	P-value
**Age <40 years old**, **men** (**n = 2**,**080**)				
Rheumatoid arthritis	1.64 (1.08–2.49)	0.021*	1.50 (0.98–2.30)	0.063
Control	1.00		1.00	
**Age <40 years old**, **women** (**n = 4**,**660**)				
Rheumatoid arthritis	1.40 (1.14–1.70)	0.001*	1.28 (1.05–1.57)	0.017*
Control	1.00		1.00	
**Age 40–59 years old**, **men** (**n = 3**,**370**)				
Rheumatoid arthritis	2.32 (1.80–2.99)	<0.001*	2.21 (1.71–2.85)	<0.001*
Control	1.00		1.00	
**Age 40–59 years old**, **women** (**n = 13**,**970**)				
Rheumatoid arthritis	1.17 (1.05–1.30)	0.003*	1.10 (0.99–1.22)	0.074
Control	1.00		1.00	
**Age ≥60 years old**, **men** (**n = 2**,**395**)				
Rheumatoid arthritis	1.55 (1.18–2.04)	0.002*	1.46 (1.11–1.93)	0.007*
Control	1.00		1.00	
**Age ≥60 years old**, **women** (**n = 7**,**000**)				

^*^Cox-proportional hazard regression model, Significance at P < 0.05.

^†^Stratified model for age, income, and region of residence, hypertension, diabetes mellitus, and dyslipidemia histories.

^‡^Adjusted model for depression histories, and Charlson Comorbidity Index.

**Table 4:** Analysis of crude and adjusted hazard ratios (95% confidence interval) of rheumatoid arthritis for asthma according the number of clinic visits of asthma.

Characteristics	Asthma			
	Crude	P-value	Adjusted^†^	P-value
**Asthma ≥3 times of clinics visit with medication histories**				
Rheumatoid arthritis	1.32 (1.23–1.44)	<0.001*	1.25 (1.16–1.36)	<0.001*
Control	1.00		1.00	
**Asthma ≥4 times of clinics visit with medication histories**				
Rheumatoid arthritis	1.38 (1.26–1.51)	<0.001*	1.30 (1.19–1.43)	<0.001*
Control	1.00		1.00	
**Asthma ≥5 times of clinics visit with medication histories**				
Rheumatoid arthritis	1.38 (1.25–1.53)	<0.001*	1.30 (1.17–1.44)	<0.001*
Control	1.00		1.00	

^*^Cox-proportional hazard regression model, Significance at P < 0.05.

^†^Stratified model for age, income, and region of residence, hypertension, diabetes mellitus, and dyslipidemia histories.

^‡^Adjusted model for depression histories, and Charlson Comorbidity Index.

**Table 5:** Crude and adjusted hazard ratios (95% confidence interval) of rheumatoid arthritis for asthma according to their treatments.

Characteristics	Asthma			
	Crude^†^	P-value	Adjusted^†‡^	P-value
**Methotrexate** (**n = 12**,**335**)				
Rheumatoid arthritis	1.32 (1.18–1.48)	<0.001*	1.27 (1.14–1.43)	<0.001*
Control	1.00		1.00	
**Leflunomide** (**n = 3**,**955**)				
Rheumatoid arthritis	1.31 (1.09–1.58)	0.004*	1.23 (1.02–1.48)	0.033*
Control	1.00		1.00	
**Other treatments** (**n = 17**,**185**)				
Rheumatoid arthritis	1.28 (1.16–1.41)	<0.001*	1.20 (1.09–1.32)	<0.001*
Control	1.00		1.00	

^*^Cox-proportional hazard regression model, Significance at P < 0.05.

^†^Stratified model for age, income, and region of residence, hypertension, diabetes mellitus, and dyslipidemia histories.

^‡^Adjusted model for depression histories, and Charlson Comorbidity Index.

## Discussion

The present study demonstrated a higher risk of asthma in rheumatoid arthritis patients than in participants in control group matched for age, sex, income, region of residence, and past medical history. The rheumatoid arthritis patients demonstrated a 1.23-times higher risk of asthma than those in the control group (95% CI = 1.15–1.32, P < 0.001). In addition, the subgroup analyses demonstrated that the increased risk of asthma was consistent according to age and sex and the medication histories of rheumatoid arthritis. This study has added to previous findings by using a control group matched for socioeconomic factors and past medical history. Moreover, various comorbidities were adjusted using CCI.

Similar to the present results, several studies have reported a relationship between asthma and rheumatoid arthritis^5,17,19^. However, the majority of previous studies were based on cross-sectional analyses, which could not demonstrate causality between asthma and rheumatoid arthritis^17,18^. A recent population-based cohort study demonstrated a 2.07-times increased risk of asthma in rheumatoid arthritis patients (95% CI = 1.99–2.15)^5^. However, they used control group matched only for age and sex. Thus, the possible influence of socioeconomic factors could not be excluded in that study. In particular, because they used medical claim code data, medical accessibility should have been unbiased between the study and control groups. However, medical accessibility might have been different according to socioeconomic status. Thus, socioeconomic factors should be considered when analyzing medical claim code data. From the present results, it can be concluded that the risk of asthma was increased in rheumatoid arthritis patients independent of socioeconomic factors, demographic factors and past medical history. The verified definitions of both asthma and rheumatoid arthritis potentiated the feasibility of the present study^3,13,22^. To improve the fidelity of the diagnosis of asthma, subgroup analyses were performed according to the number of treatment histories of asthma in this study. As results, all the asthma subgroups with ≥3, ≥4, and ≥5 times of treatment histories were elevated in rheumatoid arthritis patients.

The plausible pathophysiologic mechanisms for the increased risk of asthma in rheumatoid arthritis could be explained by immune imbalance, heterogeneous asthma characteristics, and bystander effects.

The abnormal Th1 response that occurs in rheumatoid arthritis patients could influence an uncontrolled Th2 response, which induces asthma. The counterregulation between Th1-type immunity and Th2-type immunity has been found, with some exceptions, in several studies^18,23^. Th1 and Th2 lymphocytes are classified based on their distinct cytokine secretion profiles^24^. However, Th2-type cytokines have been identified to contribute to the development of some autoimmune diseases^25,26^. In addition, an animal study demonstrated a switch from Th1 lymphocyte phenotypes from proinflammatory IFN-gamma-secreting Th1 cells to IL-4-secreting Th2 cells upon stimulation with cytokines^27^. Abnormalities in the cytokines affecting the Th1-Th2 balance may contribute to the development of asthma in rheumatoid arthritis patients. Moreover, Th17 cells have been reported to be increased in both asthma and rheumatoid arthritis patients^18^. The overlapping effects of some subsets of Th1 or Th2 cells could link rheumatoid arthritis and a high risk of asthma.

The systemic inflammatory conditions of rheumatoid arthritis could increase the risk of asthma with variety of immunologic mechanisms in addition to skewed Th2 responses. Nonallergic asthma might be caused by the inflammatory responses associated with rheumatoid arthritis. A recent microarray study demonstrated numerous novel pathways, such as those associated with neuronal function, WNT pathways, and actin cytoskeleton pathways, in asthma patients in addition to the classical type 2 inflammation-related genes^16^. Compared to childhood-onset asthma, adult asthma has several subtypes that have pathological heterogeneity^28^. Several asthma endotypes have been proposed based on distinct pathophysiologic mechanisms including nonallergic origins of noneosinophilic (neutrophilic) asthma and airflow obstruction caused by obesity^29^. Therefore, systemic inflammatory or immune dysfunctions in rheumatoid arthritis patients might lead to the several types of asthma endotypes. For instance, neutrophilic asthma could be induced due to deprivation of anti-inflammatory responses in rheumatoid arthritis patients^30^.

The medication of rheumatoid arthritis could increase the risk of asthma. The disease-modifying drugs and immunosuppressant used in rheumatoid arthritis patients were suggested to have toxic effects to lung function^31^. They reported that hypersensitivity reaction and susceptibility to infection following the use of rheumatoid arthritis medication could be predisposed the rheumatoid arthritis patients to lung diseases^31^. However, the effects of these disease-modifying drugs and immunosuppressant on airway hyperresponsiveness and asthma were controversial. A few experimental studies demonstrated the attenuation of allergen-induced airway inflammation and sensitization after long-term use of leflunomide^32,33^. In addition, immunosuppressant was suggested to relieving asthma by inhibition of T cell activation^34^. To evaluate the effects of rheumatoid arthritis medications on the risk of asthma, the present study analyzed the risk of asthma according to the types of rheumatoid arthritis medications. As results, all the types of rheumatoid arthritis medication groups showed the increased risk of asthma. Therefore, the association between rheumatoid arthritis and asthma is beyond the effects of rheumatoid arthritis medications.

The common pathophysiological causes of asthma and rheumatoid arthritis could mediate the risk of asthma in rheumatoid arthritis patients. Unidentified common comorbidities could result in both rheumatoid arthritis and asthma. Cardiovascular diseases have been reported to be related to both asthma and rheumatoid arthritis^7,35^. Likewise, diabetes also increases the risk of both rheumatoid arthritis and asthma^8,36^. Although we matched and adjusted for these comorbidities, a possible influence of these or other unconsidered confounders could not be excluded. In addition, other environmental factors including smoking, could link rheumatoid arthritis with asthma. Because the HIRA-NSC data does not include lifestyle factors, such as smoking, alcohol consumption, and body mass index, these factors could not be considered in the present study.

In the subgroup analyses performed in this study, all of the age and sex subgroups showed an increased risk of asthma in rheumatoid arthritis patients. On the other hand, a previous study demonstrated a differential association between asthma and rheumatoid arthritis according age and sex^5^. A population cohort study reported the risk of asthma in rheumatoid arthritis patients was higher in subgroups of women and younger subjects (<40 years old)^5^. Differences in medical care could result in highly detectable rates in women and the younger population included in that study.

This study based on the large, population cohort encompassing a whole nation. The current data minimized missing participants by basing the sample on the national medical registry database. In Korea, all citizens are registered and covered by the HIRA. This HIRA data was sampled by a statistician to represent the whole national population, which was verified in a previous study^13,37^. Both rheumatoid arthritis and asthma were defined by ICD-10 codes and medication history. The unbiased control group was another advantage of the present study. The control group was sampled with a random number order and matched for age, sex, income, region of residence, and past medical history of hypertension, diabetes, and dyslipidemia. Because rheumatoid arthritis is influenced by socioeconomic status, the consideration of this factor might be crucial^38^.

However, the limited information on the severity and treatment of both rheumatoid arthritis and asthma limited the interpretations of the present study. Detailed laboratory data including serum levels of autoantibodies and lung function testing could not be accessed in this study. Thus, the heterogeneous nature of rheumatoid arthritis or asthma could not be classified in this study. Lastly, the confounding effects of unavailable variables, such as smoking, alcohol consumption, obesity, diet, and nutrition, are still possible.

In conclusion, rheumatoid arthritis increased the risk of asthma in an adult Korean population. The high risk of asthma was consistent according to age and sex subgroups.

## Materials and Methods

### Study population and data collection

The ethics committee of Hallym University (2017-I102) approved the use of these data. Written informed consent was exempted by the Institutional Review Board.

This national cohort study relied on data from the Korean Health Insurance Review and Assessment Service-National Sample Cohort (HIRA-NSC). The detailed description of this data was described in our previous studies^39,40^.

### Participant selection

Of 1,125,691 cases with 114,369,638 medical claim codes, the 7,783 of rheumatoid arthritis patients were selected following the previous studies that reported the prevalence and incidence of rheumatoid arthritis of Korea (Supplement File S1)^3,22^. The 230,764 of asthma patients were selected using the modified method of a previous study (Supplement File S1)^13^.

**Figure 2:**
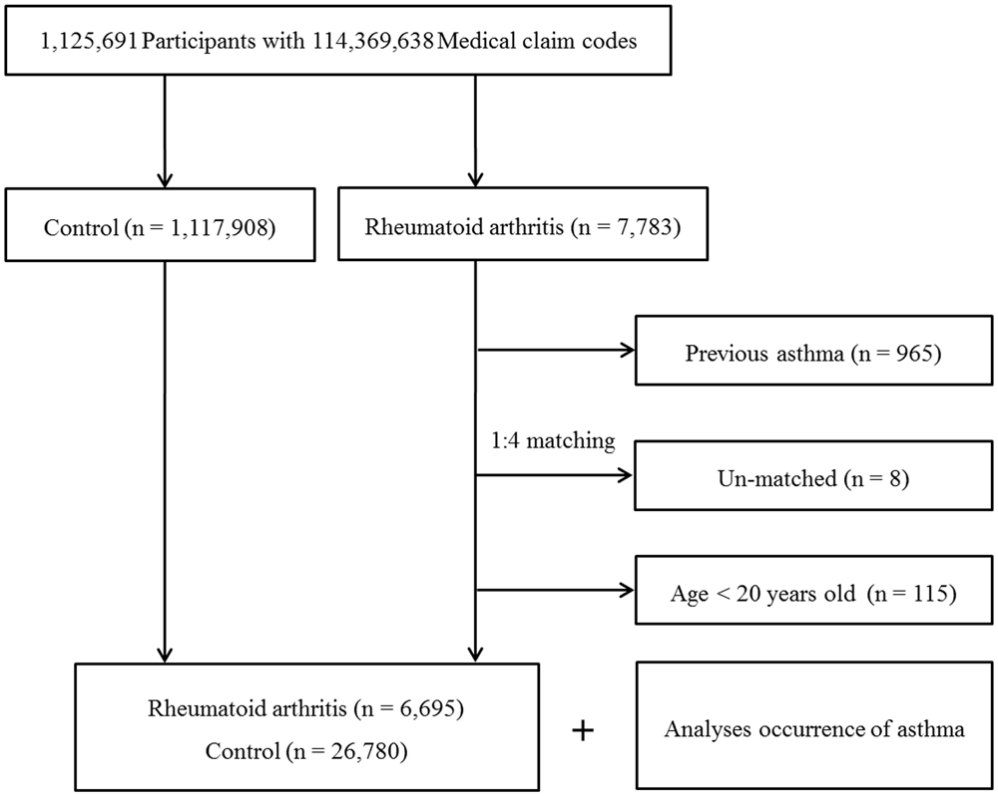
A schematic illustration of the participant selection process used in the present study. Of a total of 1,125,691 participants, 6,695 rheumatoid arthritis patients were matched with 26,780 control participants for age, group, sex, income group, region of residence, and past medical history.

The rheumatoid arthritis group was matched 1:4 with participants (control group) who were not diagnosed with rheumatoid arthritis from 2002 through 2013. The control group was selected from the total population (n = 1,117,908). Matching was performed for age, group, sex, income group, region of residence, and past medical history (hypertension, diabetes, and dyslipidemia). To prevent selection bias when selecting the matched participants, the control participants were sorted using a random number order and were then selected from top to bottom. We set the index date as the date of the diagnosis of rheumatoid arthritis. It was assumed that the matched control participants were involved at the same time as the rheumatoid arthritis participants (index date). Therefore, the control patients who died before the index date were excluded. The participants with a history of asthma before the index date were excluded from both the rheumatoid arthritis and control groups. In the rheumatoid arthritis group, 965 participants were excluded. The rheumatoid arthritis patients for whom we could not identify sufficient matching participants were excluded (n = 8). We also excluded the participants younger than 20 years of age (n = 115). The mean follow up time from index date to last date (Dec. 31, 2013) or death date was almost similar in both rheumatoid arthritis (89.5 months, SD = 43.0) and control group (90.0 months, SD = 43.2). Finally, 1:4 matching resulted in the inclusion of 6,695 rheumatoid arthritis patients and 26,780 control participants (Fig. 2).

### Variables

The age, sex, income, region of residence, hypertension, diabetes, dyslipidemia, and depression were defined as previous studies^39^. Depression was adjusted due to the previously reported association between asthma and depression^41^. CCI was used for 16 comorbidities as the continuous variable (0 [no comorbidity] through 27 [multiple comorbidities]) except for pulmonary disease and rheumatoid^42^.

### Statistical analyses

Chi-square tests were used to compare the general characteristics between the rheumatoid arthritis and control groups.

Stratified Cox proportional hazard models were used to assess hazard ratios (HRs) for rheumatoid arthritis with respect to asthma. In this analysis, crude (simple) and adjusted (for depression and CCI) models were used, and 95% CIs were calculated. In these analyses, age, sex, income, region of residence, hypertension, diabetes, and dyslipidemia were stratified. Kaplan Meier analysis and Log rank test was used.

For the subgroup analyses, we divided the participants by age (20–39, 40–59, and 60+ years) and sex (men and women) to confirm that these relations were reliable according to age and sex. For the sensitivity analyses, we analyzed the limited number of participants who were visited clinics ≥3 times, 4 times, and 5 times for asthma were included. In addition, the rheumatoid arthritis patients were classified according to the most frequently used rheumatoid arthritis medications including methotrexate, leflunomide, and other medication and analyzed the relation with the rate of asthma.

Two-tailed analyses were conducted, and P values less than 0.05 were considered to indicate significance. The results were statistically analyzed using SPSS v. 21.0 (IBM, Armonk, NY, USA).

## Supplementary information

supplement 1
