# Supplementary material for: Increased risk of asthma in patients with rheumatoid arthritis: A longitudinal follow-up study using a national sample cohort
Source: Sci Rep. 2019 May 6;9:6957. doi: 10.1038/s41598-019-43481-3 (PMC6502877; doi:10.1038/s41598-019-43481-3)
Supplement: Supplementary file 1 — supplement 1 [file 41598_2019_43481_MOESM1_ESM.docx]

**Increased risk of asthma in patients with rheumatoid arthritis: A longitudinal follow-up study using a national sample cohort**

So Young Kim^1^, Chanyang Min, PhD^2,3^, Dong Jun Oh^4^, Hyo Geun Choi^2,3*^

^1^Department of Otorhinolaryngology-Head & Neck Surgery, CHA Bundang Medical Center, CHA University, Seongnam, Korea

^2^Hallym Data Science Laboratory, Hallym University College of Medicine, Anyang, Korea

^3^Graduate School of Public Health, Seoul National University, Seoul, Korea

^3^Department of Otorhinolaryngology-Head & Neck Surgery, Hallym University College of Medicine, Anyang, Korea

^4^Department of Internal medicine, Asan Medical Center, University of Ulsan College of Medicine, Seoul, Korea

**Running head:** Asthma and rheumatoid arthritis

**Key words:** asthma; arthritis, rheumatoid; cohort studies; epidemiology

*Correspondence: [pupen@naver.com](mailto:pupen@naver.com)

**Supplement file S1 the definitions of rheumatoid arthritis and asthma**

Rheumatoid arthritis was selected using ICD-10 codes (M05 or M06) and a prescription for a biologic agent or any disease-modifying antirheumatic drug (DMARD) (n = 7,783).

We included participants who were diagnosed with asthma (ICD-10: J45) or status asthmaticus (J46) from 2002 through 2013. Among them, we selected the participants who were diagnosed with asthma by a physician more than 2 times and those who were treated with asthma-related medications including inhaled corticosteroids (ICS), ICS combined with long-acting β2-agonists (LABAs), oral leukotriene antagonists (LTRAs), short-acting β2-agonists (SABAs), systemic LABAs, xanthine derivatives, and systemic corticosteroids (n = 230,764).

**Supplement file S2 the classifications of variables**

The age groups were classified using 5-year age intervals: 20-24, 25-29, 30-34, …, and 85+ years old. A total of 14 age groups were designated. The income groups were initially divided into 41 classes (one health aid class, 20 self-employment health insurance classes, and 20 employment health insurance classes). These groups were recategorized into 5 classes (class 1 [lowest income]-5 [highest income]). The region of residence was divided into 16 areas according to administrative district. These regions were regrouped into urban (Seoul, Busan, Daegu, Incheon, Gwangju, Daejeon, and Ulsan) and rural (Gyeonggi, Gangwon, Chungcheongbuk, Chungcheongnam, Jeollabuk, Jeollanam, Gyeongsangbuk, Gyeongsangnam, and Jeju) areas.

The participants’ prior medical histories were evaluated using ICD-10 codes. To ensure an accurate diagnosis, hypertension (I10 and I15), diabetes (E10-E14), and dyslipidemia (E78) were regarded as present if a participant was treated ≥ 2 times. Depression was defined based on ICD-10 codes from F31 (bipolar affective disorder) to F39 (unspecified mood disorder) recorded by a psychiatrist ≥ 2 times.
